# CSF proteins of inflammation, proteolysis and lipid transport define preclinical AD and progression to AD dementia in cognitively unimpaired individuals

**DOI:** 10.1186/s13024-024-00767-z

**Published:** 2024-11-11

**Authors:** Marta del Campo, Carlos Quesada, Lisa Vermunt, Carel F. W. Peeters, Yanaika S. Hok-A-Hin, Calvin Trieu, Anouk den Braber, Inge M. W. Verberk, Pieter J. Visser, Betty M. Tijms, Wiesje M. van der Flier, Charlotte E. Teunissen

**Affiliations:** 1 Neurochemistry Laboratory and Biobank, Department of Laboratory Medicine, Amsterdam Neuroscience, Amsterdam University Medical Center, Vrije Universiteit Amsterdam, Amsterdam, The Netherlands; 2 Barcelonaβeta Brain Research Center (BBRC), Pasqual Maragall Foundation, Barcelona, Spain; 3 Hospital del Mar Research Institute (IMIM), Barcelona, Spain; 4 Departamento de Ciencias Farmacéuticas y de La Salud, Facultad de Farmacia, Universidad San Pablo-CEU, CEU Universities, Madrid, Spain; 5 Departmento de Matemática Aplicada a Las TIC, Polytechnical University of Madrid, Madrid, Spain; 6 Alzheimer Center Amsterdam, Department of Neurology, Amsterdam Neuroscience, Vrije Universiteit Amsterdam, Amsterdam, The Netherlands; 7 Mathematical & Statistical Methods Group (Biometris), Wageningen University & Research, Wageningen, The Netherlands; 8 Department of Biological Psychology, Vrije Universiteit Amsterdam, Amsterdam, The Netherlands; 9 Department of Epidemiology & Data Science, VU University Medical Center, Amsterdam, Netherlands

## Abstract

This preclinical AD CSF proteome study identified a panel of 12-CSF markers detecting amyloid positivity and clinical progression to AD with high accuracy; some of these CSF proteins related to immune function, neurotrophic processes, energy metabolism and endolysosomal functioning (e.g., ITGB2, CLEC5A, IGFBP-1, CST3) changed before amyloid positivity is established.

**Supplementary Information:**

The online version contains supplementary material available at 10.1186/s13024-024-00767-z.

## Main text

Pathological features of Alzheimer´s disease (AD) start to develop decades before the appearance of clinical signs, providing a unique opportunity to define and intervene AD biologically before the irreversible brain damage occurs. AD is a multifactorial disorder in which multiple factors and pathways beyond amyloid and tau pathologies are involved (e.g., immunity, lipid metabolism, vascular dysfunction, endocytic pathway) [1]. Cerebrospinal fluid (CSF) reflects the ante-mortem biochemical alterations occurring in the brain and can thus provide the pathobiological fingerprint of AD *in vivo* [2–4]. CSF proteome is dynamic and protein levels change over the AD disease stages [2, 5]. This is not trivial as a detailed analysis of the in vivo CSF proteome of cognitively unimpaired individuals in the AD preclinical stage may unveil proteins and biological pathways especially relevant for the etiology and progression of AD pathophysiology. These could be useful as biomarkers to improve the biological prognosis of AD, potential therapeutic targets and surrogate endpoints for clinical trials conducted in pre-dementia stages targeting different disease mechanisms [6, 7].

**Fig. 1:**
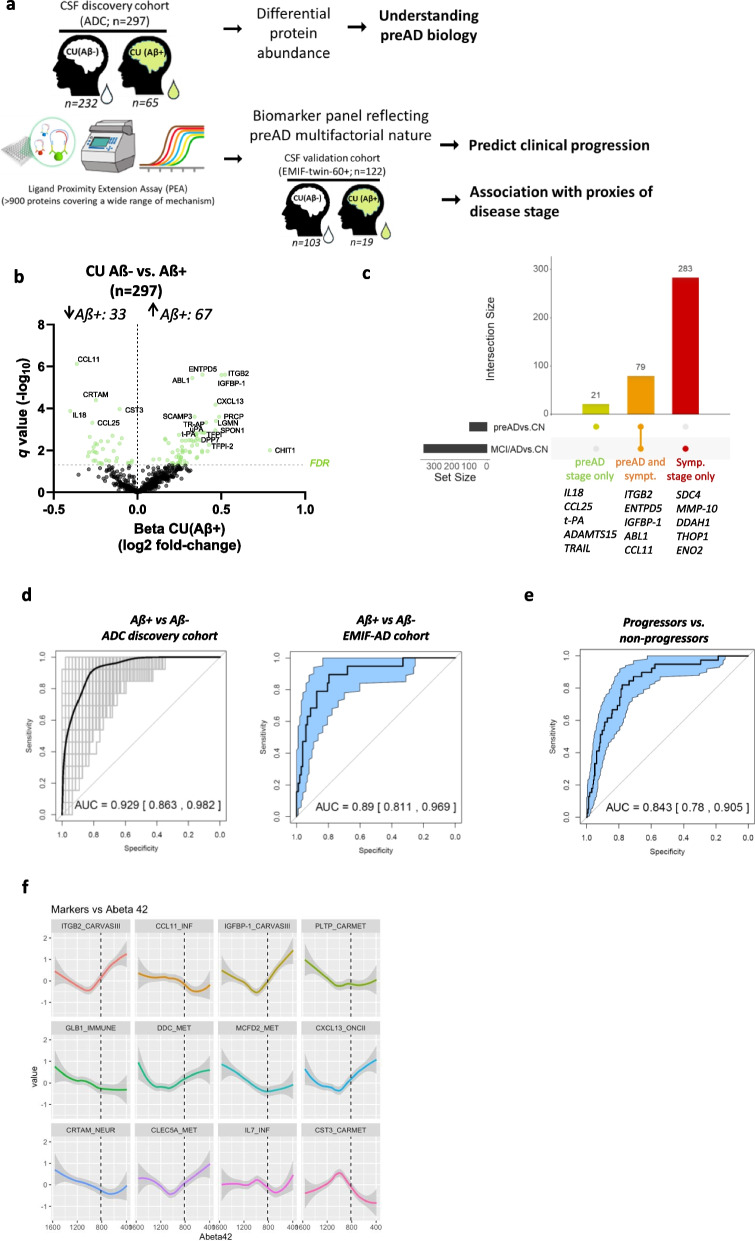
CSF proteins of inflammation, proteolysis and lipid transport define preclinical AD and predict progression to AD dementia in cognitively unimpaired individuals **a** Protein levels in CSF from cognitively unimpaired individuals with and without amyloid pathology (based on CSF Aß42 concentration; CU(Aß +) = 65; CU(Aß-) = 232) were measured using antibody-based PEA technology. We defined which proteins differed across groups and a classification model to identify preclinical AD cases, which was validated in an independent cohort of cognitively unimpaired volunteers (EMIF-AD cohort, CU(Aß-):103; CU(Aß +): 19). The performance of the model to predict clinical progression to cognitive impairment was also evaluated in a subset of cases from the discovery cohort (progressors to symptomatic stages: 39). We also evaluated the association of the proteins within the panel with proxies of progression of AD pathophysiology (e.g., CSF Aß42). **b** Volcano plot shows the CSF proteins that are differentially regulated in CU(Aß +) vs. CU(Aß-). Each dot represents a protein. The beta coefficients (log2 fold-change) are plotted versus q values (-log10-transformed FDR corrected p-value). Proteins significantly dysregulated after adjusting for false discovery rate (FDR, q < 0.05) are coloured in light green. The name of the top ten significant dysregulated CSF proteins and the top five with the strongest effect sizes are annotated. The total number of proteins that are down-regulated (left) or up-regulated (right) in CU(Aß +) is indicated. Horizontal dotted line indicates the significance threshold. Adjusted p values (q < 0.05) were calculated using a two-sided nested linear model adjusting for FDR. **c** UpSet plot indicates the overlap across the proteins regulated in the preclinical phase of AD (CU(Aß +) vs. CU(Aß-)) and those at the prodromal or dementia AD stage (MCI(Aß +) or AD (positive AD CSF profile) vs. controls) based on the results from our previous study [2]. **d** Receiver operating characteristic (ROC) curves depict the performance of 12-CSF protein panel to discriminate amyloid positive from amyloid negative cognitively unimpaired individuals in the ADC discovery and the EMIF-AD validation cohorts. In the discovery ADC cohort, black line is the mean Area Under the Curve (AUC) over all re-samplings (1000 repeats of fivefold cross-validation, grey lines). Inserts outline corresponding AUC and 95% CI. In the EMIF-AD validation cohort, insert outline the resulting AUC after directly applying the model developed with the discovery cohort. **e** ROC analysis depicts the performance of the CSF preAD panel to predict cases that progressed to MCI or dementia stage (progressors: 39; non progressors: 258). **f** CSF proteins within the preAD panel modelled along CSF Aβ42 as an early proxy of AD pathology progression. Each NPX protein value was transformed to z-scores based on the distribution in the actual dataset to allow visual comparison across proteins. Bold line indicates the mean trajectory and shadows the 95% CI. Dotted line depicts CSF Aß42 positivity threshold (< 813 pg/mL). CN, cognitively unimpaired controls; preAD: preclinical AD (CU individuals with amyloid pathology); MCI, Mild cognitive impairment; AD, Alzheimer’s disease; MMSE, Mini Mental Score Examination. Some images within Fig. 1a are courtesy of Olink® Proteomics AB

We here analyzed > 900 CSF proteins reflecting a wide range of mechanisms in the presymptomatic phase of AD (297 cognitively unimpaired individuals (CU): 232 amyloid negative and 65 amyloid positive), with 72% followed-up clinically [8, 9]. Proximity extension assay (PEA) proteome data from 122 CU participants (103 amyloid positive and 19 amyloid negative; Supplementary Material: Fig. 1) from the EMIF-AD preclinical cohort [10] was used to dismiss proteins in downstream analyses with highly divergent results across CU cohorts and to validate the biomarker panel (see Supplementary Material 1). Demographic characteristics, core AD CSF biomarker concentrations and follow-up information of the cohorts used are described in Supplementary Material 2 Table 1. Figure 1a presents an overview of the study design including the following aims: (i) define the biological changes that characterize the preclinical stage of AD; (ii) identify and validate the panel of markers needed to identify cases who are in the preclinical stage of AD and evaluate if these markers can predict clinical progression to cognitive impairment; and (iii) model the levels of these markers along the levels of CSF Aβ42 as a proxy of pathophysiological progression, and define if biomarker changes occurs before or after significant amyloidosis in the brain is established (structural break, see Supplementary Material 1).

CSF proteome profiling revealed a total of 100 unique proteins differentially regulated in amyloid positive CU compared to those with negative amyloid status (FDR < 0.05; Fig. 1b, Extended data Table 1 (ED Table 1)) after excluding those proteins that showed opposite effects in the independent EMIF-AD data set (*n* = 43 proteins, Supplementary Material 1, Supplementary Material: Table 2 and ED Table 1). The top 5 differentially regulated proteins are involved in immune function (ITGB2, CCL11), protein glycosylation and folding (ENTPD5), insulin growth factor signaling pathway (IGFBP-1) and protein phosphorylation (ABL1). CHIT1, involved in reactive gliosis and increased in different neurodegenerative dementias including AD [2], showed the strongest effects in correlation with amyloid pathology. This was followed by ITGB2, IGFBP-1, PRCP, LGMN (Fig. 1b and ED Table 1), the last two proteins are involved in lysosomal proteolytic function. The different proteins identified support the multifactorial biology of AD from the very early stages. Interestingly, 79 of the 100 unique proteins dysregulated in preclinical AD were also dysregulated in symptomatic stages of AD [2] (Fig. 1c) [2–4, 11, 12], supporting their association to AD. The fact that ITGB2 and APOL1 were the only two proteins among the top candidates previously identified at the AD dementia stage [2], underpins the importance of analyzing preclinical phases to identify relevant proteins at these earlier stages. Functional enrichment analysis showed that the 100 CSF markers dysregulated in preclinical AD were mainly associated to proteolysis and immune response (Supplementary Material: Fig. 2), pathways involved in AD pathophysiology associated with the development of amyloid and tau misfolding [1, 2]. This is in line with a recent targeted CSF proteomic study in autosomal AD showing that proteins associated with immune function were dysregulated 6 years before disease onset [5].

We next aimed to condense the proteomics data into practical biomarker signatures (minimal combination of proteins leading to the highest performance) using generalized linear modeling (GLM) with an elastic net penalty [2, 13]. Classification modelling revealed a panel of 12-CSF proteins to detect individuals in the preclinical phase of AD with an area Under the ROC curve (AUC) of 0.93, which was validated in the EMIF-AD cohort (0.89 AUC, Fig. 1d and Supplementary Material: Table 3). In line with the pathway enrichment analysis for the total regulated protein dataset, most of the proteins within the panel were mainly related to immune function (ITGB2, CXCL13, CLEC5A, CCL11, MCFD2, CRTAM, IL7). The panel also contained proteins related to dopamine biosynthesis (DDC), lysosome activity (GLB1), protease inhibition (CST3 or so-called Cystatin-C) or lipoprotein metabolism and lipid transport (IGFBP-1, PLTP). Interestingly, both CLEC5A and ITGB2 can regulate the expression or activation of TYROBP/DAP12 protein, the strongest microglia network regulator associated with sporadic late onset AD pathophysiology [14], which supports the potential role of these proteins in the earliest stages of AD pathophysiology. We know that many of these proteins (e.g., ITGB2, IGFBP-1, CLEC5A) were also specifically associated with AD when compared to a group with non-AD dementia (Supplementary Material: Fig. 3) [2], and that all markers except IL7 correlated with CSF Aβ_42_ or (p)Tau levels (Supplementary Material: Fig. 4), overall supporting their association to AD pathophysiology. We observed that the 12-CSF panel predicted progression to mild cognitive impairment (MCI) or dementia stage with 84% performance in the subset of cases followed clinically (*n* = 39 out of 213; Fig. 1e). This performance increased to 90% when predicting the group that progressed to MCI or dementia due to AD (i.e. amyloid positive only, Supplementary Material: Figure 5a), underpinning the relevance of the proteins´ panel and their mechanisms for progression to AD dementia. In line with those results, panel positivity at baseline was associated with increased risk of clinical dementia (Hazard ratio = 8.37; *p* < 0.0001; Supplementary Material: Fig. 5b). Linear mixed model analysis further showed that panel positivity was associated with a steeper cognitive decline over time as measured by mini mental score examination (MMSE, *p* = 0.01, Supplementary Material: Fig. 6), supporting the clinical relevance of the panel.

We next modelled biomarker levels along the AD pathology using CSF Aβ_42_ as the proxy of early pathological changes [6] (Fig. 1f), and evaluated at what point of amyloid load the levels of these novel biomarkers start to change (structural break; Supplementary Material 1, Supplementary Material: Fig. 7). We observed that all 12 panel proteins had only one structural break in the slope along the CSF Aβ_42_ values. Of note, all proteins except GLB1 and MCFD2 had a structural break before CSF Aβ_42_ positivity (i.e., CSF-Aβ_42_ < 813 pg/mL; Supplementary Material: Fig. 7). These results provide additional support of the relation of these proteins to the earliest detectable stages of AD and suggest that processes related to immune function, energy metabolism, neurotrophic and endolysosomal functioning start to change before significant amyloidosis is present in the brain and might thus be relevant in disease etiology. Further experimental studies are needed to understand the connection between these proteins and progression of AD pathogenesis.

Some limitations should be considered. The number of cases that converted to MCI or dementia due to AD was limited, and thus the prognostic capabilities of the panel should be validated in larger and independent cohorts with substantial follow-up clinical data. As for most CSF studies, the study was cross-sectional. Despite our results identify CSF changes associated to the earliest AD stage defined to date, future analysis with longitudinal samples covering the full AD continuum would provide a more precise picture of the temporal evolution of these different processes in sporadic AD considering also interindividual differences (e.g., basal amyloid levels, accumulation rates, resilience factors). Noteworthy, PEA proteomic technology was optimized for blood analysis and thus, different effect sizes might be obtained when using single immunoassays. Still we and others have previously validated different proteins in independent AD cohorts using custom PEA assays (e.g., those relevant for the current study such as ITGB2, DDC, CLEC5A, PARK7) [2], as well as different single immunoassays (e.g., DDC, THOP1, sTREM1, MIF, GFAP, NfL) [11–13], supporting the validity of the results obtained with PEA proteomics.

Overall, this CSF proteome profiling shows that proteins involved in immune function, proteolysis and lipoprotein metabolisms are changed before the appearance of clinical signs. A selection of this proteins can be used not only to identify preclinical AD with > 89% performance, but also to predict progression to AD dementia with good accuracy and likelihood (> 84% and HR 8.37), supporting the association of these proteins to early AD pathophysiology. Some of these proteins were abnormal even before amyloidosis is detected in CSF, suggesting that these proteins and mechanisms might be useful targets for prevention of amyloidosis and clinical symptoms due to AD. The panel identified can easily be translated into custom assays [4, 11] containing the 12 markers of interest, especially those with the strongest effect sizes (e.g., ITGB2, CCL11). This panel can be used to track in vivo processes associated with AD beyond amyloid and tau pathology before the appearance of cognitive symptoms. These results depict the multifactorial pathophysiology of AD in the earliest stages before amyloid pathology is established in vivo, providing new leads for the development of new therapeutics to counteract the development of AD and complementary biomarker tools for clinical settings and trials.

## Supplementary Information

Supplementary Material 1.

Supplementary Material 2.

Supplementary Material 3.

Supplementary Material 4.

## Data Availability

The source data generated in this study are available within this study (Extended data Table 1) or will be deposited in the synapse database under accession code https://www.synapse.org/PRIDE_SCD. The codes and scripts used in this study will be deposited in the synapse database under accession code https://www.synapse.org/PRIDE_SCD. All models were built using publicly available packages and functions in R.
