# Supplementary material for: CSF proteins of inflammation, proteolysis and lipid transport define preclinical AD and progression to AD dementia in cognitively unimpaired individuals
Source: Mol Neurodegener. 2024 Nov 11;19:82. doi: 10.1186/s13024-024-00767-z (PMC11552178; doi:10.1186/s13024-024-00767-z)
Supplement: Supplementary file 1 — Supplementary Material 1. [file 13024_2024_767_MOESM1_ESM.docx]

**Supplementary methods file**

*Participants*

An overview of the study design is presented in figure 1a. We included a total of 297 CU participants (individuals with subjective cognitive decline, SCD) from the Amsterdam dementia cohort (ADC)^1^ and the SCIENCE project^2^, of which 65 (22%) were amyloid positive based on CSF Aß42 levels (<813 pg/mL)^1,3^. A subset of these cases (n=195) were part of our previous CSF proteomic study^4^. Individuals underwent a standardized diagnostic dementia screening and SCD was assigned in a multidisciplinary meeting when individuals had subjective cognitive complaints without objective cognitive impairment at clinical and neuropsychological testing (i.e., criteria for mild cognitive impairment (MCI), dementia, or any other neurological or psychiatric disorder not fulfilled). Mini-Mental State Examination (MMSE) was used as a measure of global cognition. A total of 213 (72%) of these participants were followed on an annual basis (mean 3.4 years). At each visit, neurological and neuropsychological evaluations were repeated, and diagnosis was re­evaluated according to applicable diagnostic guidelines (e.g., MCI^5,6^, AD^7^, frontotemporal dementia -FTD-^8–10^, Vascular Dementia -VaD-^11^). 19 participants progressed to MCI, 13 progressed to AD dementia and 7 progressed to non-AD dementia (2 VaD and 5 FTD). In addition, we analysed PEA CSF proteome data available for 122 cognitively unimpaired (CU) volunteers from the Amsterdam sub-study of the EMIF-AD PreclinAD study^12^, which included 61 monozygotic twin pairs. A total of 19 (15%) participants were amyloid positive based on the ratio CSF Aß42/40 (< 0.066)^13^. We used this cohort to validate the performance of the CSF biomarker panel on detecting brain amyloidosis across cohorts of CU individuals (see statistical analysis). From the EMIF-AD cohort, only 2 cases converted to dementia stage after 6 years of follow-up and thus clinical longitudinal data from this cohort have not been included in this study.

CSF on all participants was collected by lumbar puncture and processed and stored in agreement with the JPND-BIOMARKAPD guidelines, thereby minimizing the influence of potential pre-analytical factors^14^. Levels of CSF Aβ_42_, tTau and pTau(181) (‘core AD CSF biomarkers’) were used to define amyloid positivity and to support AD diagnoses (positive CSF AD biomarker profile defined locally tTau/Aβ_42_ > 0.52)^15^. These markers were analyzed locally as part of the diagnostic procedure using commercially available kits (ADC: ELISA INNOTEST Aβ(1-42), hTAUAg, phospho-Tau(181P, Fujirebio, Ghent, Belgium) or Aβ(1-42), t-TAUAg, phospho-Tau181 Elecsys biomarker assays (Roche Diagnostics GmbH); EMIF-AD: Aβ(1-40) and Aβ(β1-42) were measured using kits from ADx Neurosciences/Euroimmun) according to the manufacturer’s instructions. Patient demographics and clinical and biochemical values from all cohorts used in this study are listed in supplementary table 1. The studies were approved by the Institutional Ethical Review Boards and informed consent was obtained from all subjects or their authorized representatives.

*CSF protein profiling*

As part of our large-scale discovery project^4^, CSF proteins (979) were quantified using the 11 specific and validated multiplex antibody-based protein panels based on the proximity extension assay (PEA) that were available at the time in which the analysis was performed (Cardiometabolic, Cardiovascular II and III, cell regulation, development, immune response, inflammation, metabolism, neurology, oncology II and organ damage; Olink Proteomics, Uppsala, Sweden)^5^. Each panel contains reagents to measure up to 92 unique proteins, though 30 proteins can be measured in several panels (replicates). Briefly, samples were randomized across plates ﻿containing appropriate intra- and inter-plate quality controls (QC) from the manufacturer and measured in three different rounds. Each round included 16 bridging samples covering different groups, which were used for reference sample normalization to control for potential batch effects. CSF samples with more than 3 QC warnings were excluded from further analysis (n=26). Each assay has an experimentally determined lower limit of detection (LOD) estimated as three standard deviations above noise level from the negative controls that are included on every plate. Only proteins with values over the lower limit of detection (LOD) in at least 85% of the samples were selected for further statistical analysis, in which remaining raw values under LOD (0.6% of all measurements) were kept as provided by manufacturer. A total of 614 proteins (599 unique proteins) in 297 CSF samples were ultimately included for statistical analysis of the discovery cohort.

*Statistical analysis & reproducibility*

All data preprocessing and analyses were conducted using R version 4.3.0 and SPSS version 25. Between-group analyses for the demographic variables were performed using two-sided one-way analysis of variance in normally distributed continuous data or Pearson’s chi-square test for categorical variables controlling for covariance when needed (e.g., age, sex). Adjustment for multiple testing was performed using Bonferroni method. Non-Gaussian distributed data were analyzed using Kruskal-Wallis Test. For the CSF proteome data, differences in protein abundance between amyloid positive and negative individuals were evaluated using nested linear models as previously described, in which for each individual protein feature, we assessed if its addition to a base model containing ﻿age and sex contributed to model fit^4,16^. Multiplicity was considered by controlling the False Discovery Rate (FDR)^17^ at *q* ≤ 0.05 based on the number of features analyzed. The same comparisons were performed in the EMIF-AD cohort and compared to those of the discovery. Proteins with highly divergent results across cohorts were removed from downstream analysis (n=43, suppl.table 2). Results were considered divergent when: i) proteins whose beta effect difference across cohorts were outliers within the distribution of beta effects of all proteins, and ii) those with beta effects higher than 0.1 or lower than -0.1 and with opposite effects across cohorts.

We next evaluated which CSF protein combination (CSF panels) best discriminated amyloid positive and negative individuals while keeping the number of markers to the minimum^4,16^. For this purpose, binary classification models were constructed by way of penalized generalized linear modeling (GLM) with an elastic net penalty (a linear combination of lasso and ridge penalties) in the discovery CSF cohort using the glmnet package and including age and sex as covariates^4,16^. This penalty enables estimation in settings where the feature to sample ratio is too high for standard generalized linear regression. Moreover, it performs automatic feature decorrelation as well as feature-selection. Unlike linear regression, this kind of modelling has a certain level of randomness and is parameter dependent so we perform 1000 repetitions of the classification and compare multiple models, showing (a) a grid of values for the elastic-net mixing parameter, reflecting strong decorrelation to a pure logistic lasso regression and (b) a grid of values providing the maximum number of proteins that may be selected under each model (21 markers maximum). The former grid (a) considers that we have little information on the collinearity burden in the data. The latter grid (b) considers that we want to keep the number of selected proteins relatively low for the future development of customized panels. The optimal penalty parameters in the penalized models were determined based on (balanced) 10-fold cross-validation of the model likelihood^4,16^. The cross-validation was performed with balanced folds, by which each fold has an outcome group ratio close to the corresponding ratio in the full data set, also referred to as stratified cross-validation. Predictive performance of all models was assessed by way of (the comparison of) Receiver Operating Characteristic (ROC) curves and Area Under the ROC Curves (AUCs). The best performance in the training phase was obtained for a model with 21 markers, however, the final selection of the panel was optimized to 12 markers using fold-based selection proportions as previously described to reduce panel complexity and avoid overfitting^4,16^. The performance (AUC) was evaluated by internal validation: repeated 5-fold cross-validation with 1000 repeats. The 95% confidence interval around the resulting AUCs was based on resampling quantiles. External validation assessed the performance of the final model with the markers of interest in the validation cohorts using ROC analysis. ROC analysis was also used to evaluate the performance of the model to identify cases that progressed to the prodromal or dementia. Sensitivity analysis was also performed to identify those cases that progress to symptomatic stages of AD only. Cox proportional hazard models adjusted for age and sex were also used to investigate the associations between panel positivity at baseline and progression to dementia. A Linear Mixed Model was fit to investigate whether panel positivity was also associated with a steeper cognitive decline over time as measured by MMSE and including age, sex and degree of education as covariates. Non-parametric correlation analysis was performed to understand the associations between the proteins within the CSF panel and the classical AD CSF biomarkers or cognitive function (MMSE score) using the complete discovery cohort without stratifying per pathological category and conditioning on age and sex as covariates.

Chow test analysis was performed to identify significant structural changes on biomarkers levels along CSF Aβ_42_ and establish at which concentration of CSF Aβ_42_ the markers start to change. Chow test is a widely utilized statistical tool to evaluate whether there are significant differences between regression models estimated over distinct segments of the data, thereby aiding in the detection of points where the data experiment a sudden change, called structural breaks^18^. Although the Chow test typically includes time as the independent variable, we here include CSF Aβ_42_ levels as the independent variable. Considering that CSF Aβ_42_ levels serve as a proxy of early AD pathology progression, results can be interpreted as the *"*evolution of markers along AD pathology" rather than the conventional "evolution of markers over time”. Structural changes in the marker’s trajectories detected for CSF Aβ_42_ values higher than the threshold for amyloid positivity may offer insights into early stages of the disease's onset.

Functional enrichment analysis was performed using Metascape^19^ selecting GO Biological Processes as ontology source. All the CSF proteins included in the final analysis were set as the enrichment background list (n=591 protein gene products). Default parameters were used for the analysis in which terms with a *p*-value < 0.01, a minimum count of 3, and an enrichment factor > 1.5 were collected and grouped into clusters based on their membership similarities.

**References supplementary methods file**

1. van der Flier, W. M. & Scheltens, P. Amsterdam Dementia Cohort: Performing Research to Optimize Care. *Journal of Alzheimer’s Disease* **62**, 1091–1111 (2018).

2. Slot, R. E. R. *et al.* Subjective Cognitive Impairment Cohort (SCIENCe): study design and first results. *Alzheimers Res Ther* **10**, 76 (2018).

3. Tijms, B. M. *et al.* Unbiased approach to counteract upward drift in cerebrospinal fluid amyloid-β 1–42 analysis results. *Clin Chem* **64**, 576–585 (2018).

4. del Campo, M. *et al.* CSF proteome profiling across the Alzheimer’s disease spectrum reflects the multifactorial nature of the disease and identifies specific biomarker panels. *Nat Aging* (2022) doi:10.1038/s43587-022-00300-1.

5. Albert, M. S. *et al.* The diagnosis of mild cognitive impairment due to Alzheimer’s disease: Recommendations from the National Institute on Aging-Alzheimer’s Association workgroups on diagnostic guidelines for Alzheimer’s disease. *Alzheimers Dement* **7**, 270–279 (2011).

6. Petersen, R. C. *et al.* Mild Cognitive Impairment: Clinical Characterization and Outcome. *Arch Neurol* **56**, 303–308 (1999).

7. Dubois, B. *et al.* Research criteria for the diagnosis of Alzheimer’s disease: revising the NINCDS-ADRDA criteria. *Lancet neurology* **6**, 734–46 (2007).

8. Rascovsky, K. *et al.* Sensitivity of revised diagnostic criteria for the behavioural variant of frontotemporal dementia. *Brain* **134**, 2456–77 (2011).

9. Armstrong, M. J. *et al.* Criteria for the diagnosis of corticobasal degeneration. *Neurology* **80**, 496–503 (2013).

10. Litvan, I. *et al.* Clinical research criteria for the diagnosis of progressive supranuclear palsy (Steele-Richardson-Olszewski syndrome): report of the NINDS-SPSP international workshop. *Neurology* **47**, 1–9 (1996).

11. Román, G. C. *et al.* Vascular dementia. *Neurology* **43**, 250–250 (1993).

12. Konijnenberg, E. *et al.* The EMIF-AD PreclinAD study: study design and baseline cohort overview. *Alzheimers Res Ther* **10**, 75 (2018).

13. Tomassen, J. *et al.* Abnormal cerebrospinal fluid levels of amyloid and tau are associated with cognitive decline over time in cognitively normal older adults: A monozygotic twin study. *Alzheimers Dement (N Y)* **8**, (2022).

14. Del Campo, M. *et al.* Recommendations to standardize preanalytical confounding factors in Alzheimer’s and Parkinson’s disease cerebrospinal fluid biomarkers: an update. *Biomark Med* **6**, 419–30 (2012).

15. Duits, F. H. *et al.* The cerebrospinal fluid ‘alzheimer profile’: Easily said, but what does it mean? *Alzheimer’s and Dementia* **10**, 713-723.e2 (2014).

16. del Campo, M. *et al.* CSF proteome profiling reveals biomarkers to discriminate dementia with Lewy bodies from Alzheimer´s disease. *Nat Commun* **14**, 5635 (2023).

17. Benjamini, Y. & Hochberg, Y. Controlling the False Discovery Rate: A Practical and Powerful Approach to Multiple Testing. *Journal of the Royal Statistical Society B* vol. 57 289–300 Preprint at https://doi.org/10.2307/2346101 (1995).

18. Chow, G. C. Tests of Equality Between Sets of Coefficients in Two Linear Regressions. *Econometrica* **28**, 591 (1960).

19. Zhou, Y. *et al.* Metascape provides a biologist-oriented resource for the analysis of systems-level datasets. *Nat Commun* **10**, 1–10 (2019).
