# Supplementary material for: CSF proteins of inflammation, proteolysis and lipid transport define preclinical AD and progression to AD dementia in cognitively unimpaired individuals
Source: Mol Neurodegener. 2024 Nov 11;19:82. doi: 10.1186/s13024-024-00767-z (PMC11552178; doi:10.1186/s13024-024-00767-z)
Supplement: Supplementary file 4 — Supplementary Material 4. [file 13024_2024_767_MOESM4_ESM.pptx]

## Slide 1
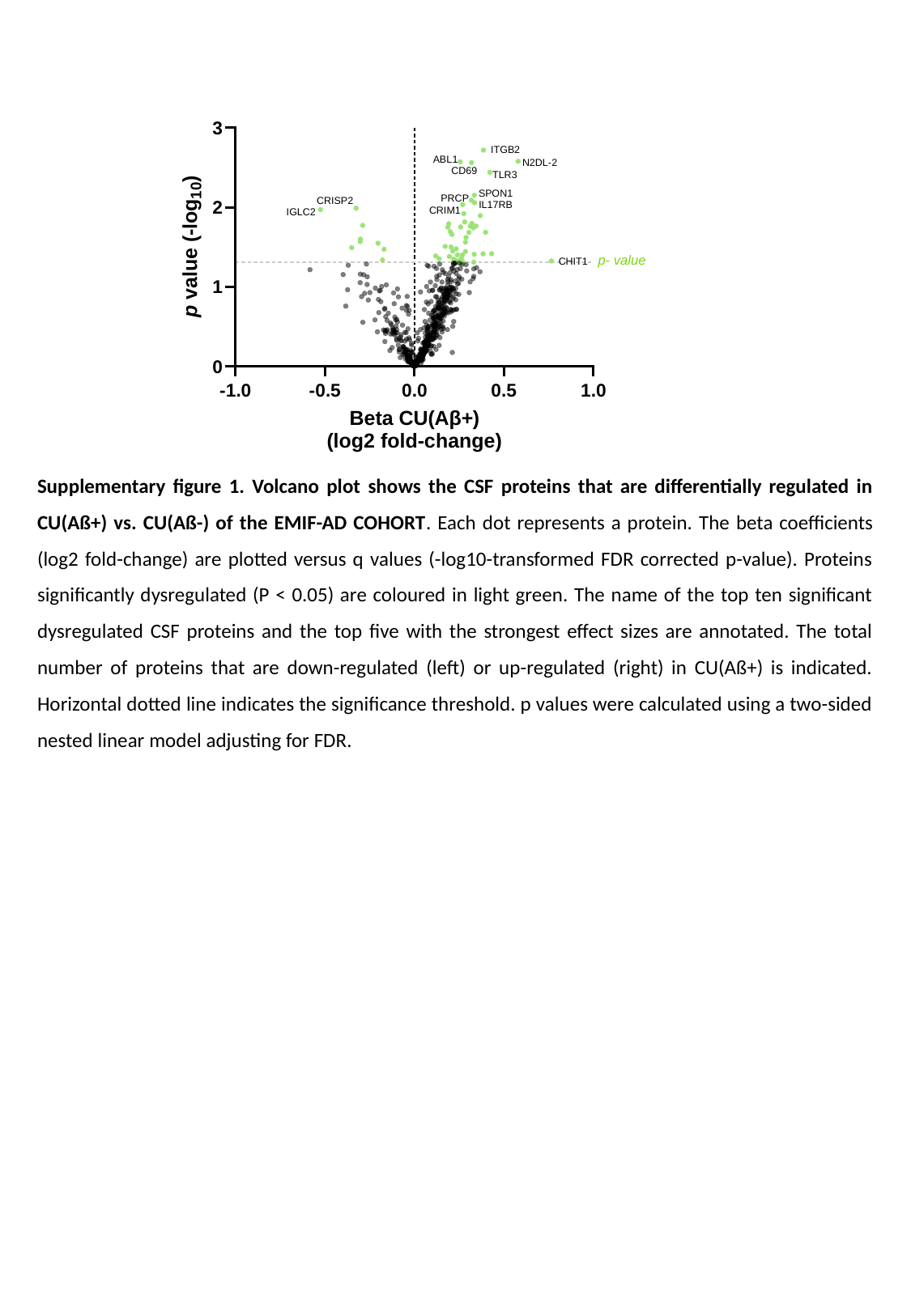

Supplementary figure 1. Volcano plot shows the CSF proteins that are differentially regulated in CU(Aß+) vs. CU(Aß-) of the EMIF-AD COHORT. Each dot represents a protein. The beta coefficients (log2 fold-change) are plotted versus q values (-log10-transformed FDR corrected p-value). Proteins significantly dysregulated (P < 0.05) are coloured in light green. The name of the top ten significant dysregulated CSF proteins and the top five with the strongest effect sizes are annotated. The total number of proteins that are down-regulated (left) or up-regulated (right) in CU(Aß+) is indicated. Horizontal dotted line indicates the significance threshold. p values were calculated using a two-sided nested linear model adjusting for FDR.

## Slide 2
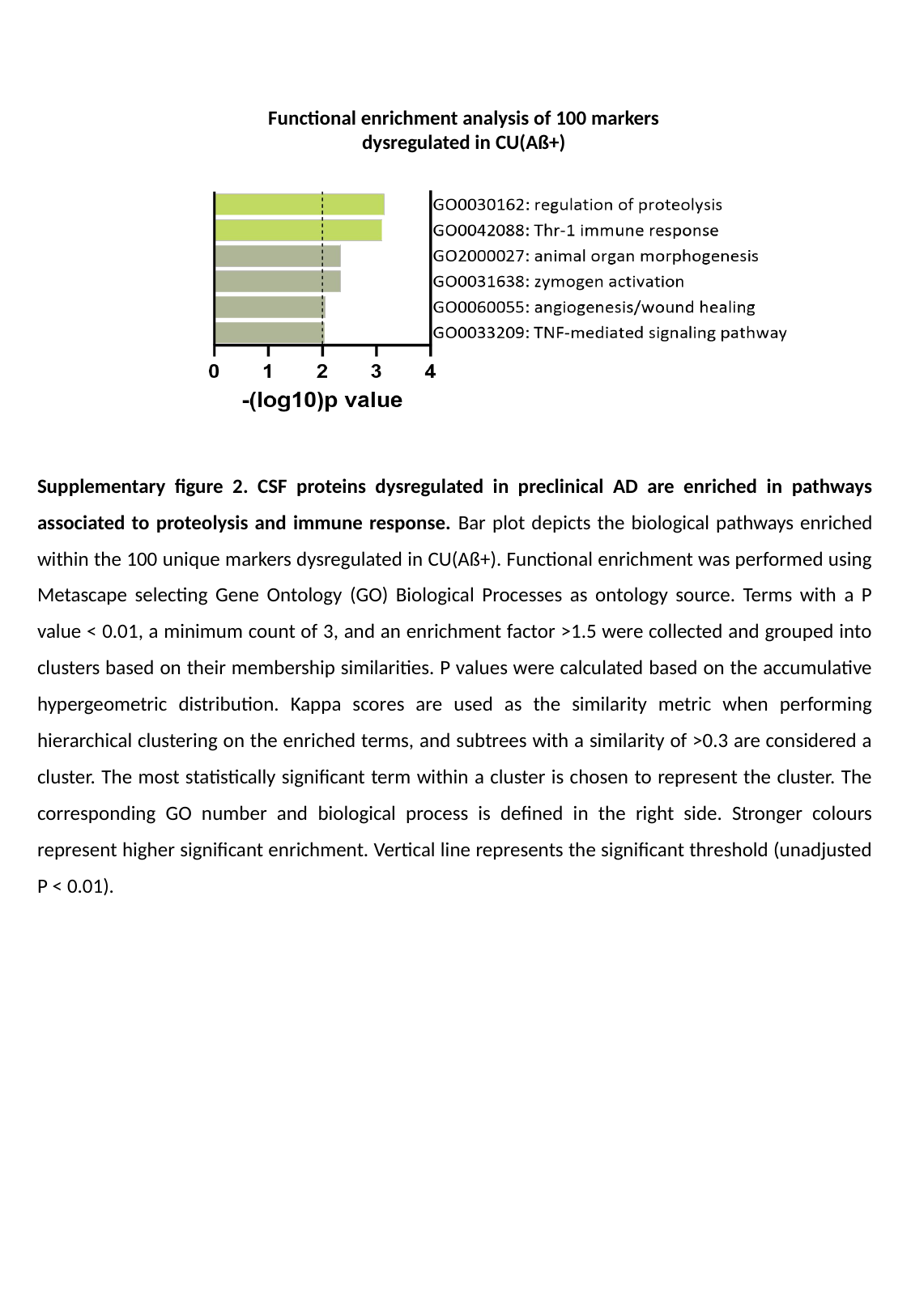

Functional enrichment analysis of 100 markers dysregulated in CU(Aß+)
Supplementary figure 2. CSF proteins dysregulated in preclinical AD are enriched in pathways associated to proteolysis and immune response. Bar plot depicts the biological pathways enriched within the 100 unique markers dysregulated in CU(Aß+). Functional enrichment was performed using Metascape selecting Gene Ontology (GO) Biological Processes as ontology source. Terms with a P value < 0.01, a minimum count of 3, and an enrichment factor >1.5 were collected and grouped into clusters based on their membership similarities. P values were calculated based on the accumulative hypergeometric distribution. Kappa scores are used as the similarity metric when performing hierarchical clustering on the enriched terms, and subtrees with a similarity of >0.3 are considered a cluster. The most statistically significant term within a cluster is chosen to represent the cluster. The corresponding GO number and biological process is defined in the right side. Stronger colours represent higher significant enrichment. Vertical line represents the significant threshold (unadjusted P < 0.01).

## Slide 3
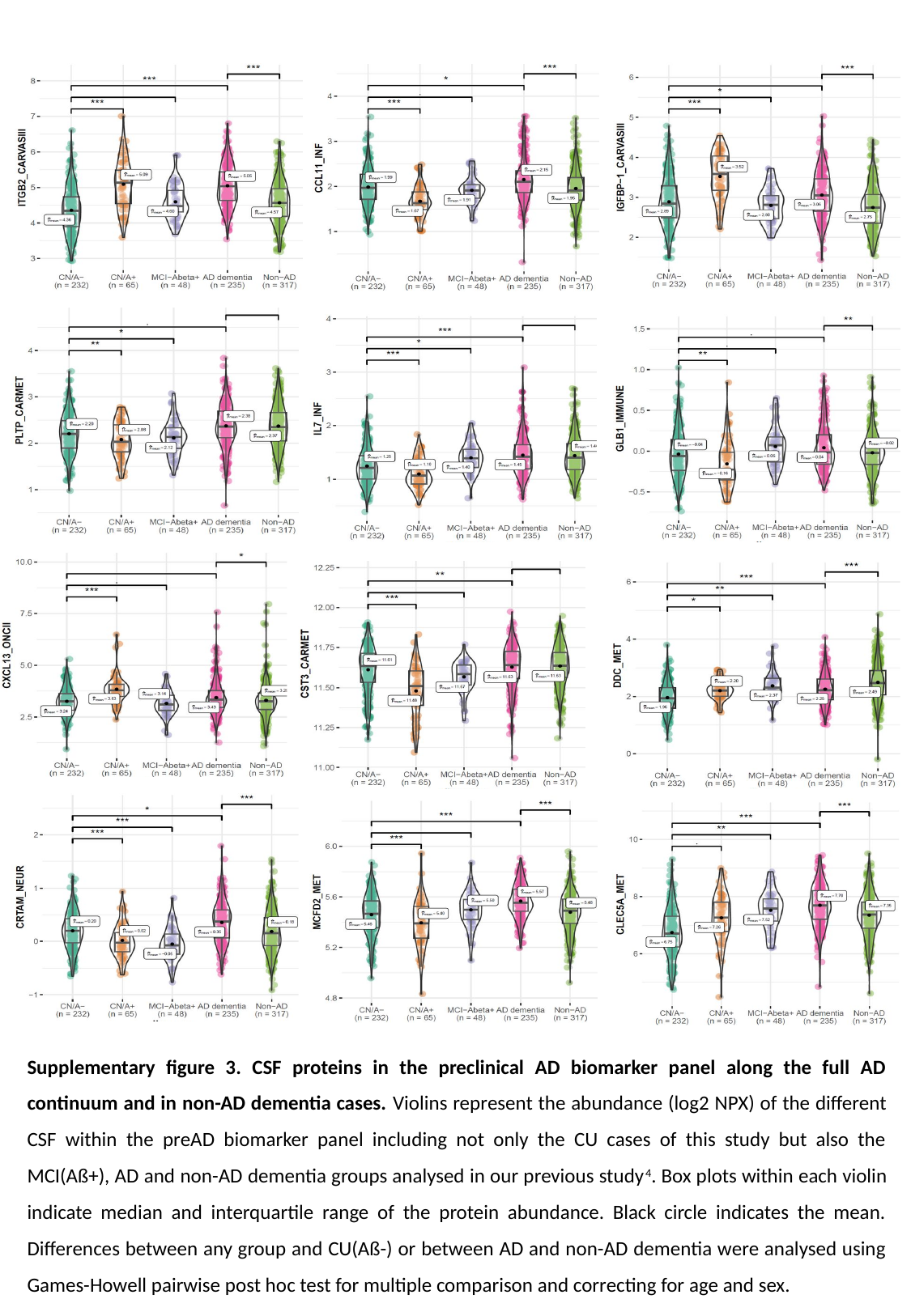

Supplementary figure 3. CSF proteins in the preclinical AD biomarker panel along the full AD continuum and in non-AD dementia cases. Violins represent the abundance (log2 NPX) of the different CSF within the preAD biomarker panel including not only the CU cases of this study but also the MCI(Aß+), AD and non-AD dementia groups analysed in our previous study4. Box plots within each violin indicate median and interquartile range of the protein abundance. Black circle indicates the mean. Differences between any group and CU(Aß-) or between AD and non-AD dementia were analysed using Games-Howell pairwise post hoc test for multiple comparison and correcting for age and sex.

## Slide 4
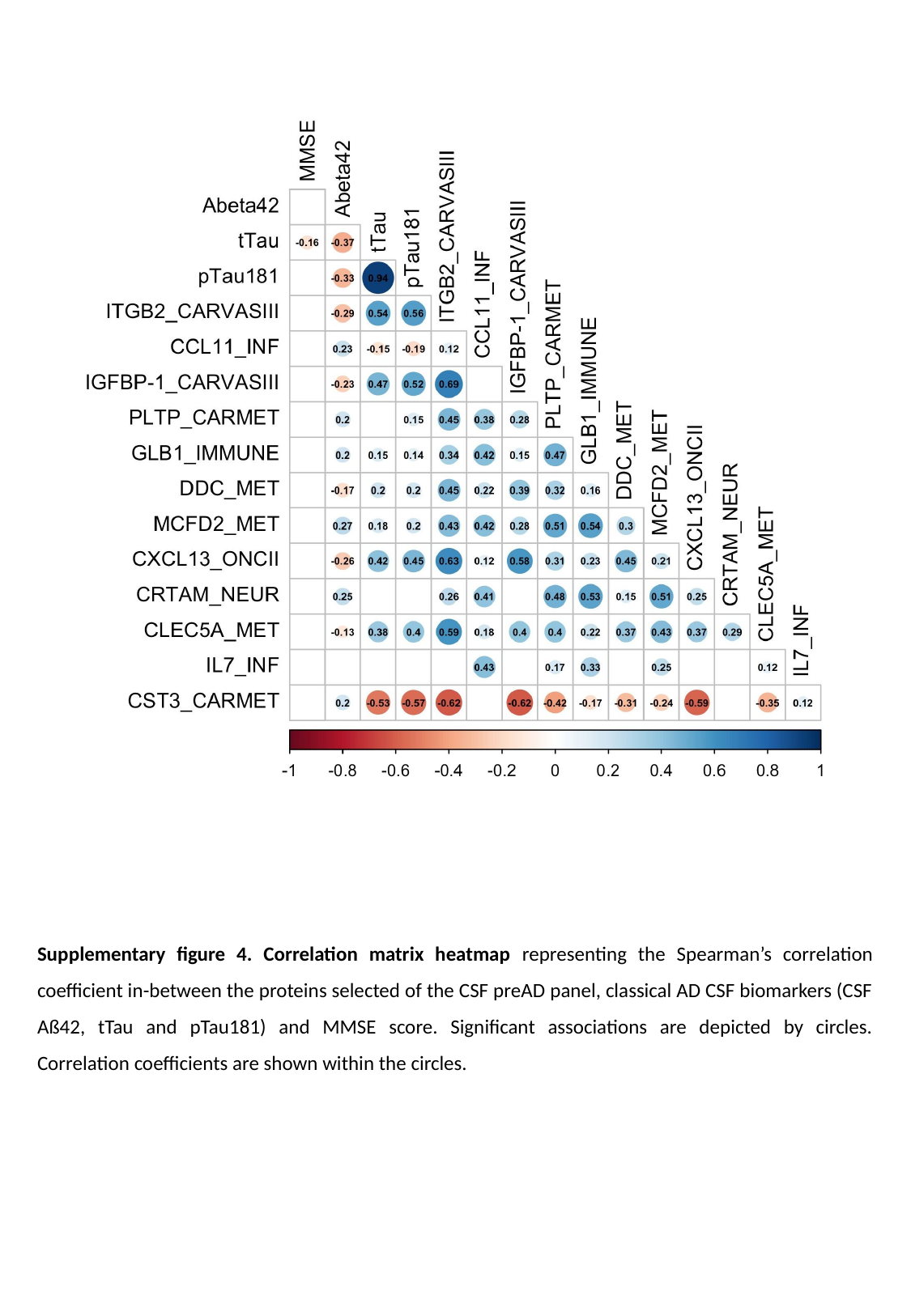

Supplementary figure 4. Correlation matrix heatmap representing the Spearman’s correlation coefficient in-between the proteins selected of the CSF preAD panel, classical AD CSF biomarkers (CSF Aß42, tTau and pTau181) and MMSE score. Significant associations are depicted by circles. Correlation coefficients are shown within the circles.

## Slide 5
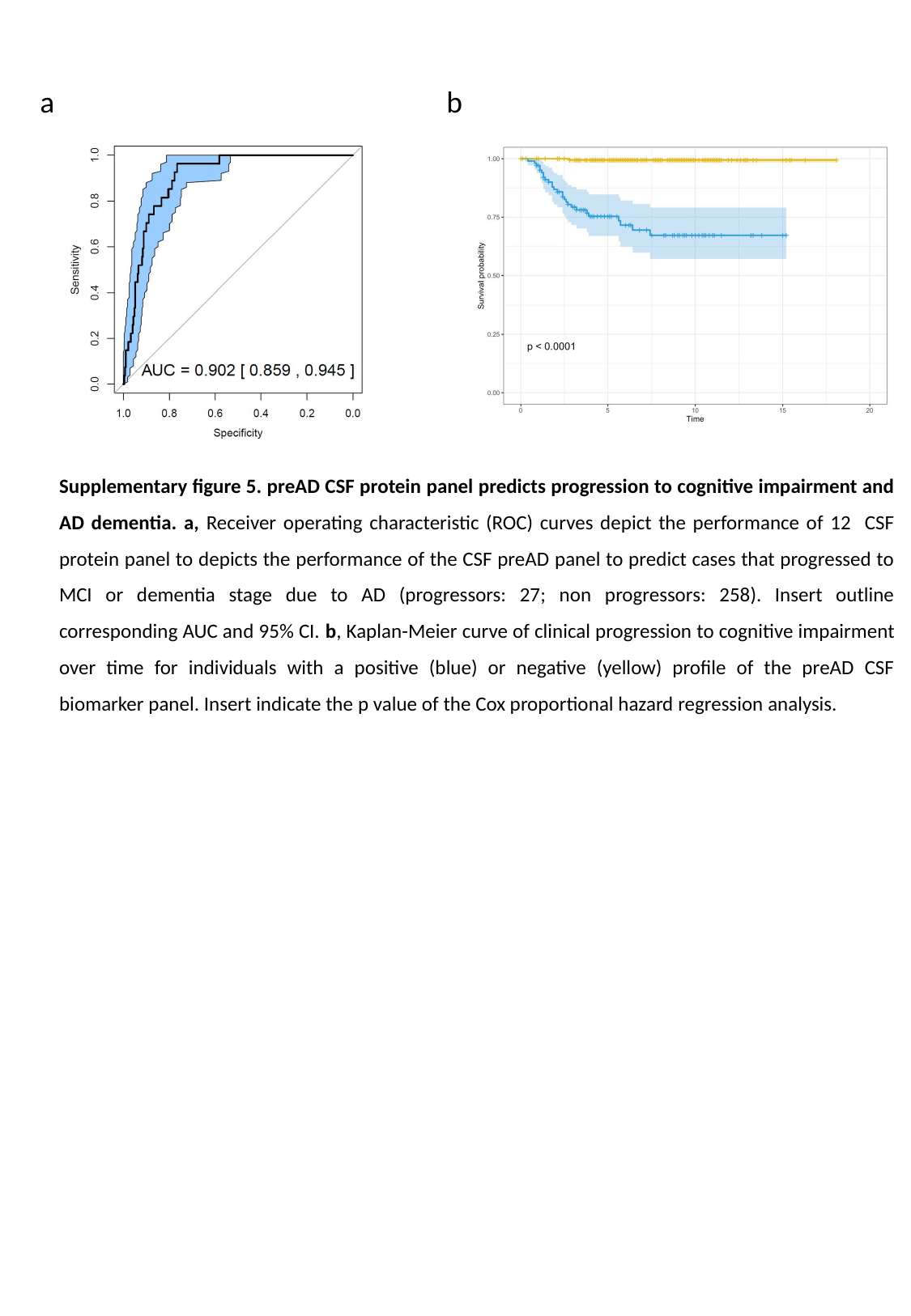

a
b
Supplementary figure 5. preAD CSF protein panel predicts progression to cognitive impairment and AD dementia. a, Receiver operating characteristic (ROC) curves depict the performance of 12 CSF protein panel to depicts the performance of the CSF preAD panel to predict cases that progressed to MCI or dementia stage due to AD (progressors: 27; non progressors: 258). Insert outline corresponding AUC and 95% CI. b, Kaplan-Meier curve of clinical progression to cognitive impairment over time for individuals with a positive (blue) or negative (yellow) profile of the preAD CSF biomarker panel. Insert indicate the p value of the Cox proportional hazard regression analysis.

## Slide 6
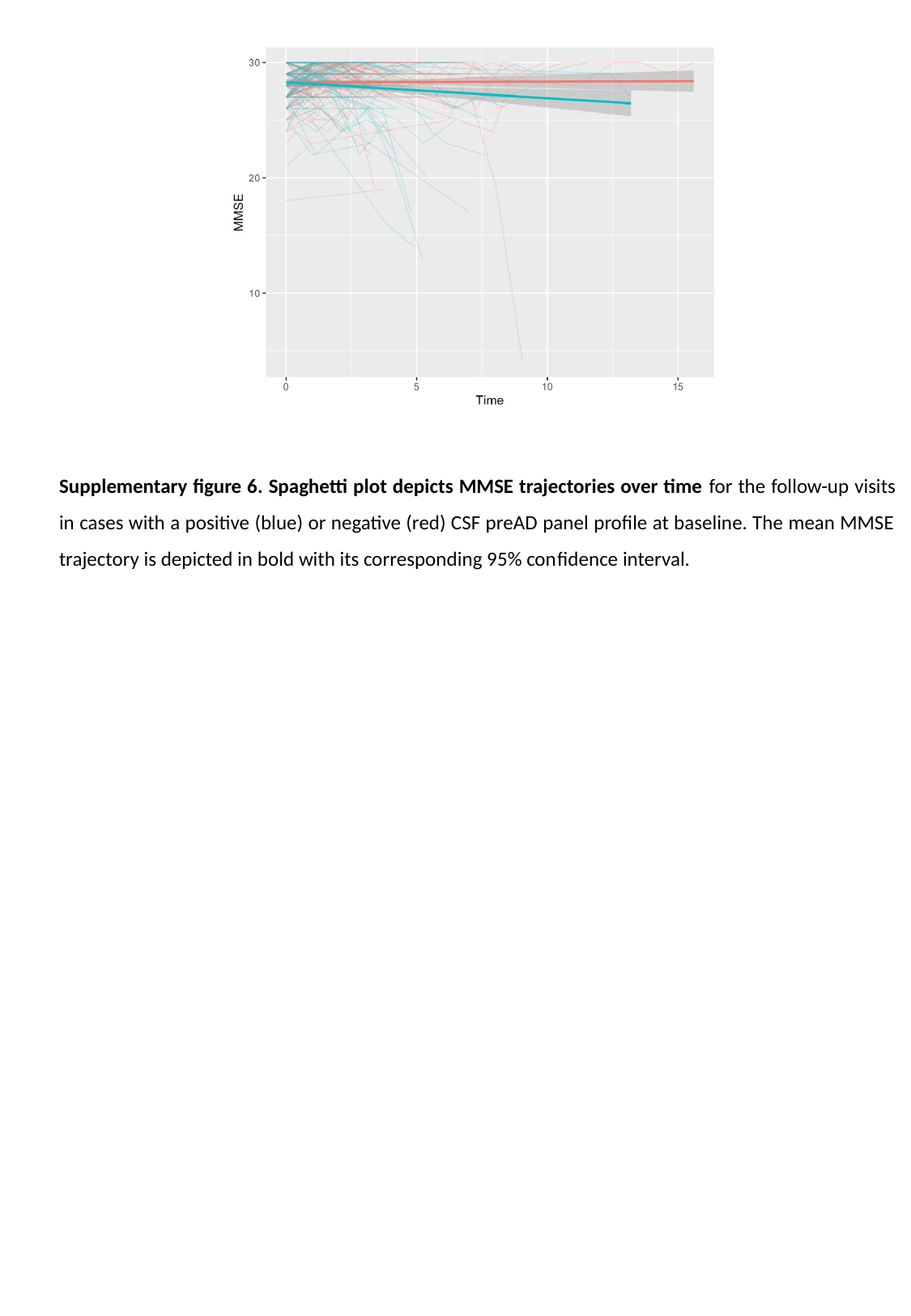

Supplementary figure 6. Spaghetti plot depicts MMSE trajectories over time for the follow-up visits in cases with a positive (blue) or negative (red) CSF preAD panel profile at baseline. The mean MMSE trajectory is depicted in bold with its corresponding 95% conﬁdence interval.

## Slide 7
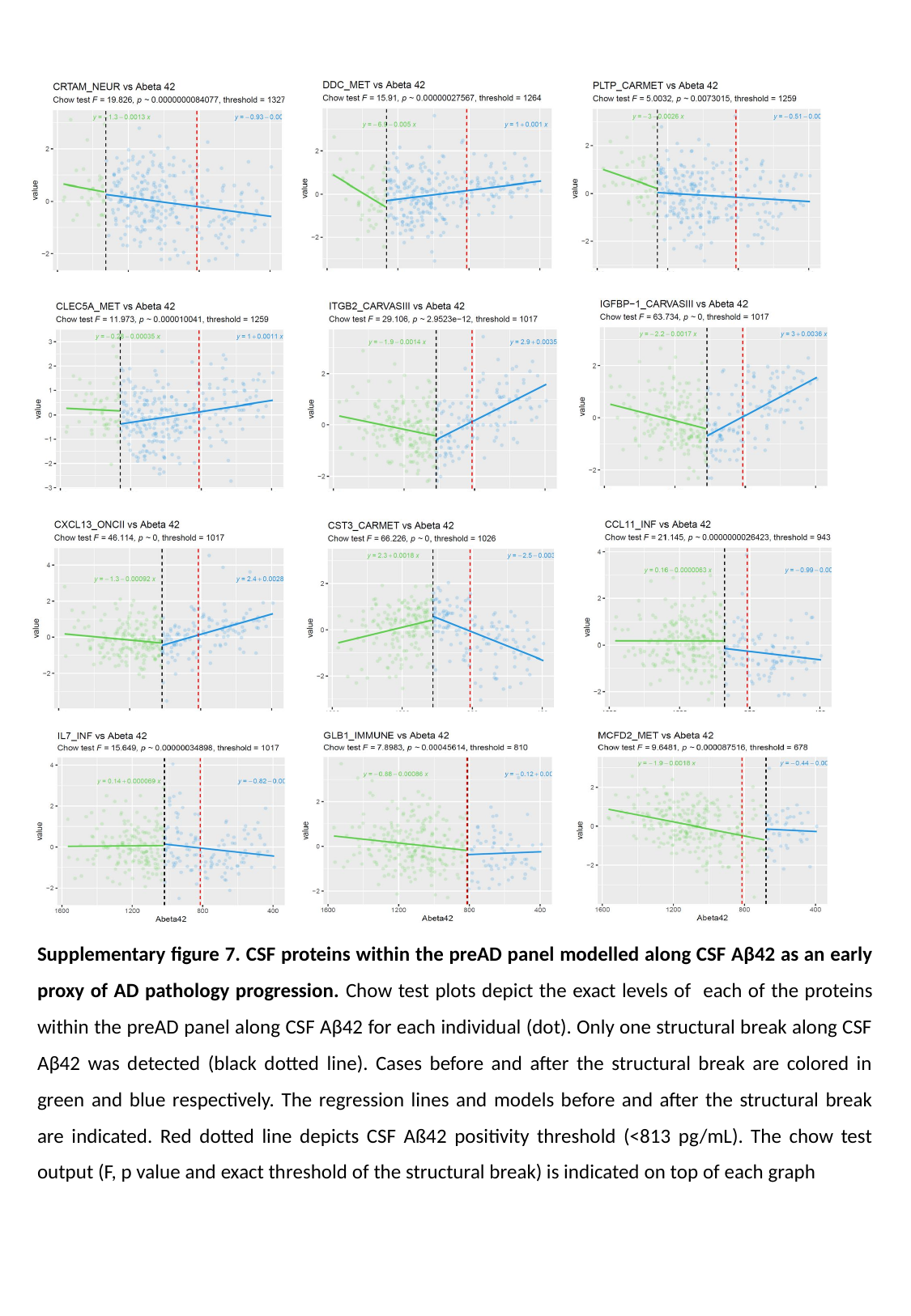

Supplementary figure 7. CSF proteins within the preAD panel modelled along CSF Aβ42 as an early proxy of AD pathology progression. Chow test plots depict the exact levels of each of the proteins within the preAD panel along CSF Aβ42 for each individual (dot). Only one structural break along CSF Aβ42 was detected (black dotted line). Cases before and after the structural break are colored in green and blue respectively. The regression lines and models before and after the structural break are indicated. Red dotted line depicts CSF Aß42 positivity threshold (<813 pg/mL). The chow test output (F, p value and exact threshold of the structural break) is indicated on top of each graph
